# Vaginal microbiome composition in pregnant and non-pregnant women: community structure, population variation, clinical impact, and metagenomics approaches

**DOI:** 10.1128/iai.00542-25

**Published:** 2026-06-15

**Authors:** Daiki Jonouchi, Srusti Shenoy, Renise Saintlouis, Amar Singh, Dharambir Kashyap, Chandana Bhargavi, Raymond Mansoor, Edmond Mansoor, Prasanna Honnavar

**Affiliations:** 1 Basic Medical Science, American University of Antigua College of Medicine, St. Johns, Antigua and Barbuda; 2 Brown Center for Immunotherapy, Melvin and Bren Simon Comprehensive Cancer Center, Division of Hematology and Oncology, School of Medicine, Indiana University 828416, Indianapolis, Indiana, USA; 3 Department of Anatomy and Medical Imaging, American University of Antigua College of Medicine, St. Johns, Antigua and Barbuda; 4 Mansoor Medicals, St. Johns, Antigua and Barbuda; 5 Department of Clinical Medicine, American University of Antigua College of Medicine, St. Johns, Antigua and Barbuda; 6 Department of Microbiology and Immunology, American University of Antigua College of Medicine, St. Johns, Antigua and Barbuda; University of California San Diego School of Medicine, La Jolla, California, USA

**Keywords:** vaginal microbiome, pregnant, metagenomics

## Abstract

The vaginal microbiome plays a critical role in reproductive health and undergoes characteristic remodeling during pregnancy that influences maternal and neonatal outcomes. Although the non-pregnant vaginal microbiome shows substantial inter-individual variability, pregnancy is associated with reduced microbial diversity and increased dominance by *Lactobacillus* species, creating a protective environment for fetal development. Disruption of this balance, termed vaginal dysbiosis, has been linked to adverse obstetric and neonatal outcomes. This narrative review synthesizes current evidence on pregnancy-associated vaginal microbiome dynamics, with emphasis on community state types (CSTs), gestational changes, population-specific variation, and clinical implications. We review studies that use 16S rRNA sequencing, next-generation sequencing, and shotgun metagenomics to characterize microbial composition across pregnancy and the postpartum period. *Lactobacillus*-dominated communities, particularly those dominated by *Lactobacillus crispatus*, are consistently associated with microbiome stability and favorable pregnancy outcomes, whereas high-diversity anaerobic communities (CST IV) are linked to bacterial vaginosis, preterm birth, miscarriage, gestational diabetes mellitus, and infection-related complications. The vaginal microbiome composition varies significantly across racial, ethnic, and geographic populations. African-descended populations more often show *L. iners*-dominant or diverse anaerobic profiles, whereas European populations more commonly show *L. crispatus* dominance. Future longitudinal and mechanistic studies across diverse populations are needed to establish causality and evaluate microbiome-based interventions to improve maternal and neonatal health.

## INTRODUCTION

The human vaginal microbiome plays a critical role in maintaining women’s reproductive health and influencing neonatal outcomes (1–3). It consists of a complex community of bacteria, fungi, and viruses that coexist in dynamic balance (4, 5). Disruptions in this balance have been associated with adverse reproductive and pregnancy outcomes such as preterm birth, bacterial vaginosis, and increased susceptibility to sexually transmitted infections (6). Previous studies across North America, Europe, and Asia have characterized the vaginal microbiota primarily using 16S rRNA gene sequencing and pyrosequencing, which primarily capture the bacterial component of the microbiome (1, 7). However, these methods often overlook other important microbial members such as fungi (mycobiome) and viruses (virome), as well as functional gene expression profiles that can influence host–microbe interactions (5). Despite global progress in microbiome research, there remains a significant gap in understanding the vaginal microbial composition. Geographic, ethnic, and environmental factors can influence microbiome structure and function, making it essential to study diverse populations (1, 2). Moreover, metagenomic and metatranscriptomic approaches such as shotgun sequencing enable comprehensive profiling of microbial diversity, functional potential, and antimicrobial resistance genes (8).

**Fig 1:**
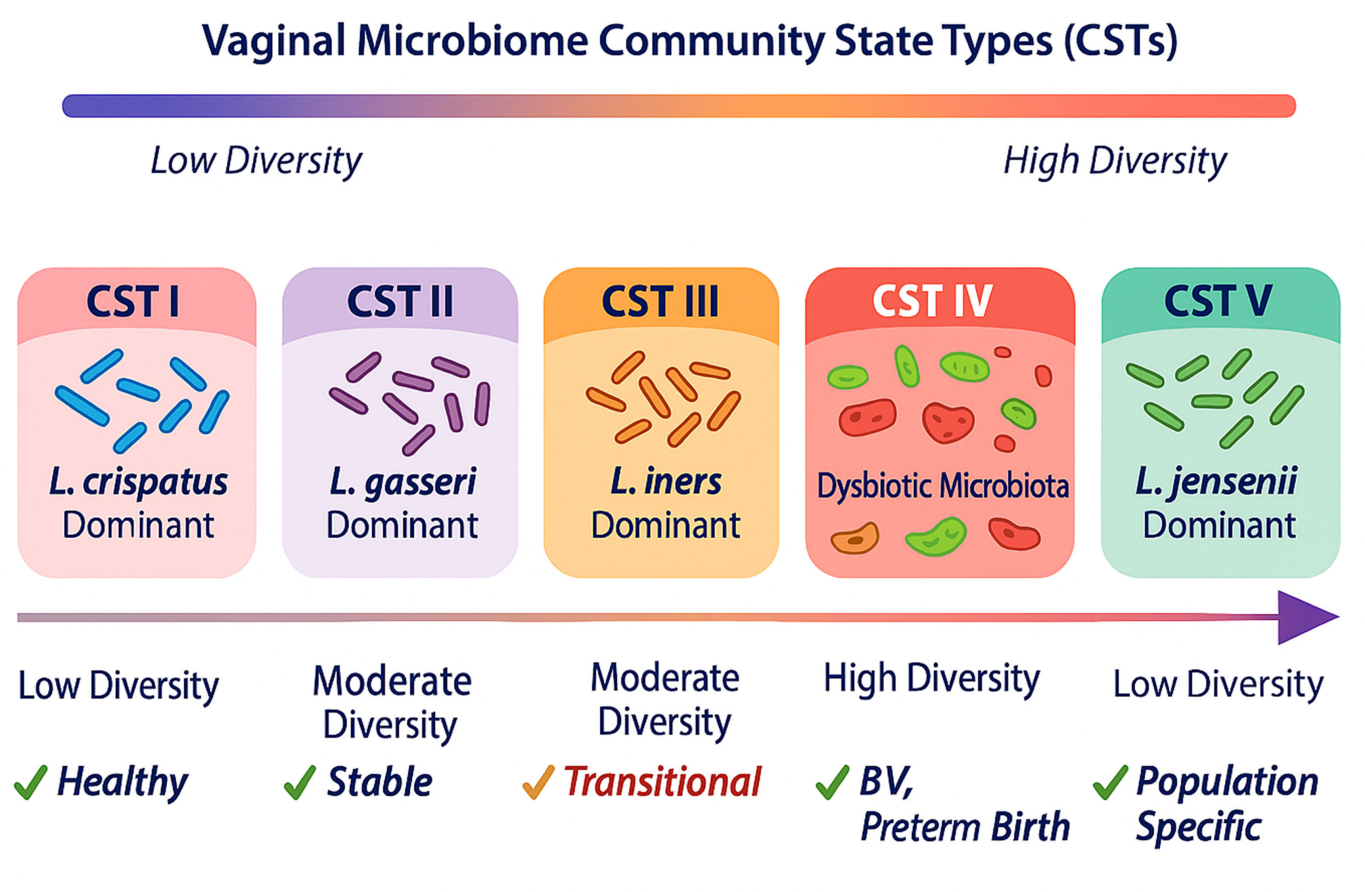
Vaginal microbiome CSTs and associated diversity patterns: The vaginal microbiome is commonly classified into five CSTs based on dominant bacterial taxa and overall microbial diversity. CST I is dominated by *Lactobacillus crispatus* and is characterized by low diversity and a stable, protective vaginal environment. CST II is dominated by *Lactobacillus gasseri* and similarly exhibits low diversity and relative stability. CST III is dominated by *Lactobacillus iners* and represents a more transitional state with moderate diversity and increased susceptibility to microbial shifts. CST IV is characterized by high microbial diversity and dominance by anaerobic taxa, including *Gardnerella, Prevotella, Sneathia*, and *Atopobium*, and is commonly associated with vaginal dysbiosis, bacterial vaginosis, and adverse pregnancy outcomes such as preterm birth. CST V is dominated by *Lactobacillus jensenii* and is observed less frequently, with prevalence varying across populations. The gradient indicates increasing microbial diversity from *Lactobacillus*-dominated to dysbiotic community structures.

The vaginal microbiota has long been studied and is associated with high-risk pregnancies (1, 2). At higher magnification, four main *Lactobacillus* species are essential for competing with other potentially pathogenic bacteria in the vagina. *Lactobacillus* helps maintain the acidic environment of the vagina, thereby promoting a healthy pregnancy and minimizing maternal complications (9). These *Lactobacillus* species include *L. crispatus, L. iners, L. gasseri*, and *L. jensenii*. The vaginal microbiome is commonly categorized into five community state types (CSTs), each defined by dominant taxa and microbial diversity patterns (Fig. 1). CST I is *L. crispatus* dominant, CST II is *L. gasseri* dominant, CST III is *L. iners* dominant, CST IV is dominated by anaerobic bacteria such as *Aerococcus, Atopobium, Dialister, Gardnerella, Megasphaera, Prevotella,* and *Sneathia*, and CST V is *L. jensenii* dominant (9). Furthermore, these subdivisions reveal differences in *Lactobacillus* CST dominance by race and ethnicity. CST I is associated with lower rates of preterm birth among individuals of European ancestry (Northern and Western Europe), as well as among Korean populations (10). In fact, in the limited number of cases with miscarriages and preterm births within these populations, their microbiota was either found to be CST III or CST IV (10). Women with African ancestry, whether in the Caribbean, the United States, Canada, or Europe, have higher rates of premature birth or other complications. These women were found to have a microbiota dominated by CST IV (11). CST IV is more biodiverse, which can hinder healthy vaginal flora (Fig. 1). Other anaerobic bacteria, such as those previously listed, will raise the pH from 3 to a more alkaline level, thereby outcompeting the vital *Lactobacillus* and endangering mother and baby. This community group is also associated with bacterial vaginosis (BV), which is a dysbiotic condition characterized by depletion of protective *Lactobacillus* species and overgrowth of anaerobic bacteria, including *Gardnerella, Mobiluncus, Bacteroides,* and *Mycoplasma hominis* (12). In addition, other BV-associated taxa, such as *Prevotella* and *Sneathia*, contribute to microbial diversity, inflammatory responses, and adverse reproductive outcomes (13, 14). The microbial imbalance is accompanied by an elevation in vaginal pH and the formation of a polymicrobial biofilm, mainly by *Gardnerella vaginalis*, which facilitates colonization by other anaerobic organisms and contributes to dysbiosis (12, 15). These changes disrupt the normal mucosal barrier, promote inflammatory responses, and facilitate ascending infection. Consequently, BV strongly associates with adverse pregnancy outcomes, such as preterm premature rupture of membranes, intra-amniotic infection, preterm birth, and neonatal complications such as respiratory distress syndrome (12, 16–18). Beyond BV-associated dysbiosis, pregnancy itself has been shown to cause a shift in the vaginal microbiome, especially in women of African descent (11). The stage at which this change occurs can also affect fetal development, and similar shifts have been reported in Hispanic populations (11). As shown in Fig. 1, differences in CST distribution across populations may contribute to variations in pregnancy outcomes, and this paper examines evidence linking these community states to unfavorable maternal and neonatal outcomes. PCR amplification of 16S rRNA genes and pyrosequencing were used for all the studies included here and are unique to bacteria, allowing further classification of *Lactobacillus* groups.

This literature review aims to comprehensively examine differences in the vaginal microbiome between pregnant and non-pregnant women, with a particular focus on studies that use next-generation sequencing (NGS) and shotgun metagenomics.

## METHODOLOGY

Titles and abstracts were initially screened for relevance by four reviewers. Eligible articles were then subjected to full-text review. Each article was independently assessed by two reviewers selected from the four-member team. Any disagreements were resolved through discussion. If necessary, a third reviewer was consulted until consensus was reached. Data were extracted manually. Because this review is narrative, a formal PRISMA flow diagram was not generated.

### Search strategy

In this review paper, we conducted a literature search across multiple databases. We used the keywords “vaginal microbiome,” “pregnancy,” and “16s RNA” to identify relevant studies. We included both primary and review papers that focus on the population structure of the vaginal microbiome and on comparisons between pregnant and non-pregnant women.

### Inclusion and exclusion criteria

Our inclusion criteria for study selection were as follows: (i) the study was conducted in humans, (ii) it focused on a comparative analysis of the microbiome between pregnant and non-pregnant individuals, (iii) microbial profiling was performed using 16S rRNA or shotgun sequencing, and (iv) details for each sample, including health and environmental status, were clearly described. Our exclusion criteria were as follows: (i) use of non-human or *in vitro* samples, (ii) insufficient sample size, (iii) the method of analysis was not fully or clearly described, and (iv) the paper was not written in English.

### Data extraction

From each study that meets our inclusion criteria and does not meet exclusion criteria, we extract the year of research or publication, country and region, characteristics of the sample population, sample size, research design, analytical technology used, significant findings, and any clinical correlation with the observation.

## VAGINAL MICROBIOME ALTERATIONS

**Fig 2:**
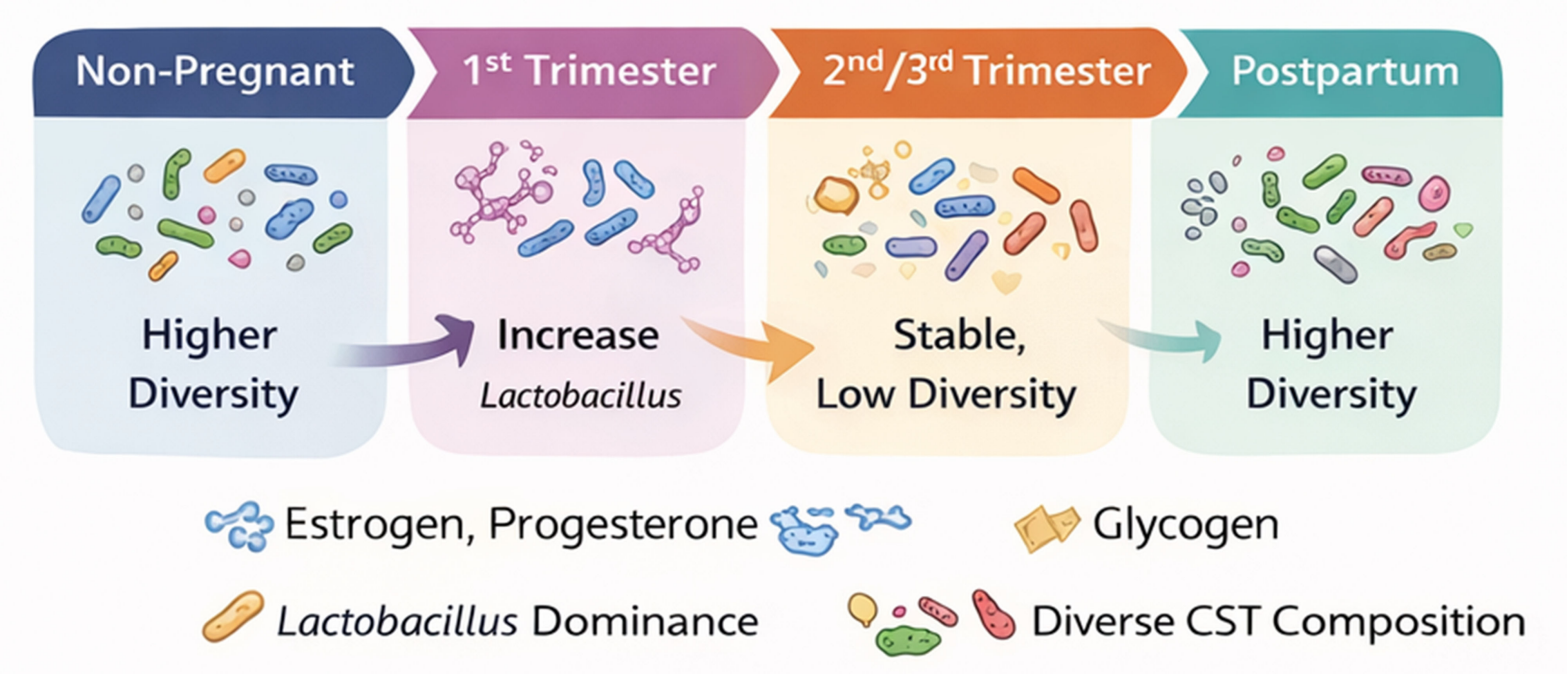
Pregnancy-associated temporal shifts in the vaginal microbiome. Pregnancy is associated with dynamic but structured changes in vaginal microbiome composition across gestation. In the non-pregnant state, the vaginal microbiome typically exhibits higher bacterial diversity with variable dominance of *Lactobacillus* species. During the first trimester, rising levels of estrogen and progesterone promote increased glycogen availability, facilitating the expansion of *Lactobacillus* spp. As pregnancy progresses into the second and third trimesters, the vaginal microbiome becomes increasingly stable, dominated by *Lactobacillus*, and less diverse, creating a more protective environment. Following delivery, postpartum hormonal shifts are associated with a return toward higher microbial diversity and reduced *Lactobacillus* dominance.

Pregnancy is associated with structured temporal shifts in vaginal microbiome composition across gestation (Fig. 2). Estrogen changes, bacterial density, and especially glycogen abundance are necessary for pregnancy-associated shifts in *Lactobacillus* spp. These shifts are associated with healthy pregnancy outcomes, as seen among Canadian, British, and American women of European descent (19). Higher estrogen levels lead to greater glycogen deposits, which are the main nutrient source for *Lactobacillus* spp. (19). As gestational age increases, bacterial vaginosis, *Ureaplasma*, and *Mollicutes* are outcompeted (19). Thus, gestational age increases linearly with *Lactobacillus* abundance and reduces bacterial biodiversity. In the postpartum stage, the microbiome returns to greater bacterial diversity and is less rich in *Lactobacillus* spp. Women of African ancestry experience a shift in *Lactobacillus* spp. dominance later in the first trimester than women of non-African ancestry (11). In women of African descent, the transitional shift is to *L. iners*, with this species increasing and directing all other bacterial shifts (11). By contrast, in the first trimester, women of non-African ancestry have higher *L. crispatus* and *L. iners*. This dominance continues to increase throughout the remainder of their pregnancy. When *L. crispatus* directs transitions or shifts in the dominant bacteria, this leads to stability and consistency, whereas changes directed by *L. iners* or *G. vaginalis* lead to more dysbiosis in the vaginal microbiomes, with frequent transitional shifts (11). These inconsistencies increase the biodiversity of *Prevotella, Gardnerella vaginalis*, and *Sneathia amnii*, all of which are associated with preterm birth, sexually transmitted infections, pelvic inflammatory diseases, and BV (11). By the second trimester, *Lactobacillus* dominance becomes more equal across racial groups, but the first trimester of pregnancy drives transitions and shifts in the dominant vaginal microbiome (11). Table 1 shows a comparison of vaginal microbiome characteristics in pregnant versus non-pregnant women.

**TABLE 1:** Comparison of vaginal microbiome characteristics in pregnant versus non-pregnant women

Feature	Non-pregnant	Pregnant
Microbial diversity	Higher	Reduced
*Lactobacillus* abundance	Variable	Increased
CST stability	Less stable	More stable
Dominant species	Mixed	*L. crispatus*, *L. iners*
Inflammatory profile	Higher	Suppressed

## METHODOLOGICAL ADVANCES IN METAGENOMICS

Advances in metagenomic analysis technology have closely driven research on the vaginal microbiome in pregnant and nonpregnant women. Initially, 16S rRNA-based metagenomics was the gold standard. For example, Zhou et al. used this method in 2007 to identify eight supergroups of the vaginal microbiome (20). The 16S rRNA sequencing process involves two main steps: terminal restriction fragment length polymorphism (T-RFLP) and clone library sequencing. T-RFLP profiles the microbial community by treating 16S rRNA sequences with restriction enzymes such as *Msp*I and *Hha*I, which highlight community characteristics. Clone library sequencing determines species by sequencing the entire 16S rRNA gene. These methods provide a general view of community structure and possible species, but their data can be unreliable.

Building on these initial techniques, NGS addressed the limitations of earlier approaches. In 2014, Fettweis et al. assessed differences in vaginal microbiome populations by nationality and race using NGS (2). The primary technological advance over the earlier study by Zhou et al. is NGS’s greater per-species sensitivity (2, 20). Unlike previous methods, NGS-based approaches typically target the variable region of the 16S rRNA gene, enabling high-resolution taxonomic profiling and generating tens of thousands of reads in a short time frame (21, 22). This approach allows detailed community analysis and detection of rare species. While highly effective for holistic bacterial analysis, NGS is limited because it cannot capture other components of the microbiome, such as fungi and viruses, and it does not provide information on specific sequences outside predefined regions (21). With these advances in place, researchers recognized the need for broader analytical techniques. According to Dunlop et al., analyzing bacteria alone is insufficient to understand the relationship between vaginal health and the microbiome, as conditions such as STIs can also be caused by viruses and parasites (23). To address this gap, shotgun sequencing emerged as a method for randomly sampling the entire DNA. This approach offers several advantages over 16S rRNA sequencing, including the detection of single-nucleotide polymorphisms, resistomes, virulent genes, and fungi. Despite drawbacks such as low cost-effectiveness and high complexity, combining NGS and shotgun sequencing could identify modifiable risk factors and potentially lead to significant improvements in vaginal health (23). Table 2 summarizes the advantages and limitations of each sequencing approach (24–26).

**TABLE 2:** Methodological comparison of sequencing approaches

Approach	Coverage	Advantage	Limitation
16S rRNA amplicon sequencing	Targets the 16S ribosomal RNA gene Resolution up to the genus level	Cost-effective Established pipelines for data analysis Large archival data sets available for reference	Results are based only on operational taxonomic units (OTUs) Limited precision at the species level No direct functional gene sequencing available
Shotgun metagenomic sequencing	Sequences total DNA Enables species- and strain-level resolution	Allows discrimination between different strains within the same species Direct functional gene identification High taxonomic precision	Relatively high cost High computational requirements The large number of strains within a single species can complicate interspecies association analysis
Multi-omics (integrated approaches)	Integrates metagenomics with microbiome profiling, transcriptomics, proteomics, and metabolomics	Systems-level integration across multiple omics dimensions Enables the construction of gene regulatory networks Facilitates identification of regulatory and causal relationships Supports host–microbiome interaction modeling	Lack of standardized analytical frameworks Increased risk of false positives High computational and bioinformatic complexity High cost and difficulty in harmonizing heterogeneous data sets

## CLINICAL IMPLICATIONS AND HEALTH OUTCOMES

### Gestational diabetes mellitus

Understanding how diabetes during pregnancy alters the vaginal microbiota is crucial for a comprehensive analysis of any possible complications. Due to the dysregulation of glucose levels, this significantly poses a threat to both the mother and the fetus. Studies have shown that women with gestational diabetes mellitus (GDM) possess less diverse vaginal microbiota, characterized by a significant depletion of *Lactobacillus* spp. (beneficial during pregnancy) and are replaced by opportunistic and pathogenic bacteria (27).

A longitudinal investigation of the vaginal microbiome in GDM reveals that the trajectory of microbial changes from late pregnancy to the postpartum differs between women with GDM and those without GDM: specifically, women with GDM show a decline in *Lactobacillus* abundance, indicating a more severe imbalance in postpartum microbial recovery (28). This is evident in that GDM alters the vaginal microbiome by dramatically reducing the protective microbiome. The decrease in this microbiota is caused by hyperglycemia and insulin resistance, which create a vaginal environment conducive to dysbiosis. High glucose levels could nourish pathogenic bacteria, allowing them to form numerous colonies. This can thereby impair mucosal barrier function (15).

Based on prior studies, the perinatal and postpartum persistence of microbial imbalance caused by GDM raises concerns about long-term vaginal health. Women with GDM are at an elevated risk of persistent dysbiosis after delivery, which can predispose them to recurrent infections and other gynecologic complications (28). It is crucial for further studies to be conducted to identify other pathologies associated with microbiome alterations in pre- and postpartum mothers with gestational diabetes mellitus.

### Pregnancy complications

**Fig 3:**
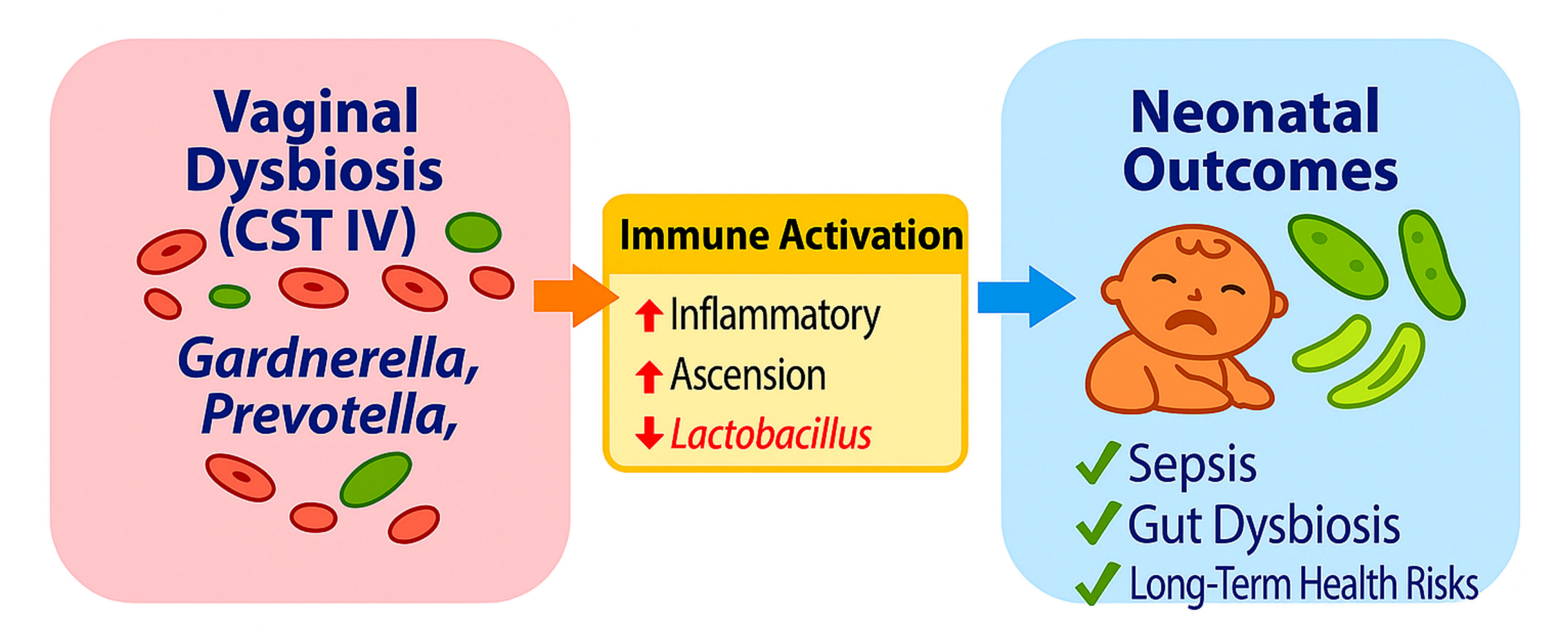
Pathway linking vaginal dysbiosis to pregnancy complications and neonatal outcomes: Vaginal dysbiosis, commonly characterized by high-diversity anaerobic communities (CST IV), is associated with inflammatory and immune dysregulation in the vaginal environment. Overgrowth of taxa such as *Gardnerella, Prevotella, Sneathia*, and *Atopobium* can promote ascending bacterial colonization, amplify inflammatory signaling, and disrupt mucosal barrier integrity. These processes contribute to pregnancy complications, including preterm birth, miscarriage, gestational diabetes mellitus, and infection-related outcomes. Altered maternal vaginal microbiota also influences neonatal health by affecting microbial seeding at birth, increasing susceptibility to neonatal sepsis and gut dysbiosis, and potentially leading to long-term immune and metabolic consequences.

Extensive studies have shown that an altered vaginal microbiome during pregnancy is associated with numerous complications, including preterm birth and infection-related outcomes (29, 30). The studies reviewed highlight the role of a *Lactobacillus*-dominated vaginal microbiome in fostering a healthy pregnancy and, conversely, show that vaginal dysbiosis is linked to adverse pregnancy outcomes (1, 30–32) (Fig. 3).

In a Korean cohort, spontaneous abortion and miscarriage were significantly associated with dysbiotic community state types (CST III and CST IV). This suggests that early variations in microbial structure can serve as prognostic factors for identifying high-risk pregnancy (10). Similarly, a study in Iran found a strong correlation between vaginal dysbiosis and spontaneous abortion (9). These findings further reinforce the relationship between microbial instability and early pregnancy loss.

Emergency cesarean section is another clinically significant complication associated with vaginal dysbiosis. In a Danish prospective study, vaginal dysbiosis occurring in early pregnancy was selectively associated with emergency, not elective, cesarean section. This was likely due to a higher incidence of fetal distress, chorioamnionitis, or labor dystocia in dysbiotic pregnancies (33). This significant observation underscores the importance of microbiome assessments to accurately inform intrapartum risk profiles. Table 3 shows pregnancy complications associated with vaginal microbiome dysbiosis.

**TABLE 3:** Pregnancy complications associated with vaginal microbiome dysbiosis

Condition	Microbiome features	Key taxa	Key reference
Preterm birth	High diversity, CST IV	*Gardnerella*, *Sneathia*	(10)
Miscarriage	CST III/IV	*Prevotella*	(15)
GDM	↓ Lactobacillus	Mixed anaerobes	(27)
Emergency C-section	Early dysbiosis	Multiple	(33)

Previous research has shown a significant link between dysbiosis and adverse infection-related outcomes. Comparative studies conducted in African, Asian, European, and mixed-ancestry populations show significant variation in the prevalence of *Lactobacillus* spp. Yet, they demonstrate that high diversity is associated with unfavorable outcomes (34). However, pregnant women with abnormal vaginal microbial states exhibit higher risks of Group B *Streptococcus* (GBS) colonization. This is a major cause of neonatal sepsis (35). These shifts in the microbiome during pregnancy suggest altered immune regulation in dysbiotic states, increasing vulnerability to infection-driven complications (36).

### Neonatal health outcomes

Alterations in the maternal vaginal microbiome have direct consequences for neonatal health, particularly through associations with preterm birth and early-life microbial colonization (37, 38). Dysbiotic vaginal communities enriched in anaerobic taxa, including *Gardnerella, Prevotella*, *Sneathia,* and *Atopobium*, have been repeatedly linked to spontaneous preterm birth, likely mediated by inflammatory signaling, membrane weakening, and ascending infection pathways (31, 39, 40). These inflammatory cascades can induce premature cervical ripening and rupture of membranes, increasing neonatal morbidity and mortality (40). In addition to influencing gestational length, the maternal vaginal microbiome plays a critical role in shaping the neonatal microbiome during vaginal delivery (41, 42). Infants born to mothers with *Lactobacillus*-dominated vaginal communities are more likely to acquire beneficial pioneer taxa, whereas neonates exposed to dysbiotic vaginal microbiota show altered gut and mucosal microbial profiles. These early microbial differences have been linked to increased susceptibility to neonatal sepsis, respiratory distress, and impaired immune development (31, 43–45).

Emerging evidence suggests that early-life microbiome perturbations may have long-term health consequences, including increased risks of allergic disease, metabolic dysfunction, and immune dysregulation (46, 47). Thus, pregnancy-associated vaginal dysbiosis is not only a risk factor for adverse birth outcomes but also a potential determinant of lifelong health trajectories.

Collectively, these findings suggest that pregnancy-associated hormonal shifts interact with host immune regulation and microbial metabolism to favor *Lactobacillus* dominance, while failure of this transition promotes inflammation-driven obstetric and neonatal complications. Table 4 shows neonatal outcomes associated with maternal vaginal microbiome composition.

**TABLE 4:** Neonatal outcomes associated with maternal vaginal microbiome composition

Neonatal outcome	Maternal microbiome profile	Proposed mechanism
Preterm birth	CST IV, anaerobic dominance	Inflammation, membrane weakening
Neonatal sepsis	Dysbiosis	Altered immune priming
Low birth weight	Reduced *Lactobacillus*	Placental inflammation
Altered infant microbiome	Dysbiosis	Abnormal microbial seeding

## POPULATION-SPECIFIC CONSIDERATIONS

The vaginal microbiome exhibits substantial variation across different populations, influenced by both intrinsic genetic factors and extrinsic environmental exposures. Understanding these population-specific patterns is critical for interpreting associations between the microbiome and pregnancy outcomes, and for developing targeted interventions for diverse populations.

### Geographic and ethnic variations

#### Racial and ethnic differences in microbial community composition

**Fig 4:**
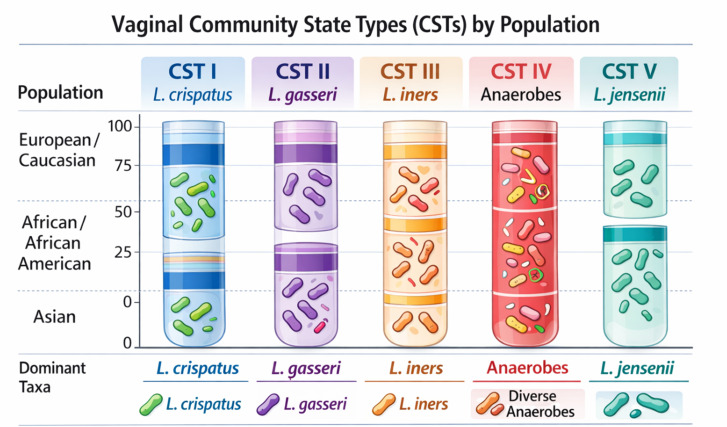
Population-specific differences in vaginal microbiome composition and community state type distribution: Vaginal microbiome composition varies substantially across populations, with distinct distributions of CSTs among racial and ethnic groups. European and Caucasian populations are more often characterized by *Lactobacillus crispatus*-dominated communities (CST I), which are associated with a lower risk of adverse pregnancy outcomes. In contrast, African and African American populations more commonly exhibit *Lactobacillus iners*-dominated (CST III) or diverse anaerobic communities (CST IV). Asian populations show greater representation of CST V (*Lactobacillus jensenii*-dominant) in some cohorts.

Accumulating evidence shows that vaginal microbiome composition varies significantly across racial and ethnic groups, with distinct taxonomic signatures that persist throughout pregnancy (Fig. 4). These differences are characterized by variation in both dominant bacterial species and overall community diversity.

#### *Lactobacillus* species distribution across populations

The distribution of *Lactobacillus* species, considered protective during pregnancy, shows marked racial and ethnic variation. European and Caucasian populations typically have higher frequencies of *L. crispatus*-dominated communities, which have been consistently linked to a lower risk of preterm birth (PTB) (48). In a large, nested case-control study, each one-log increase in normalized *L. crispatus* abundance corresponded to a 19% reduction in the multivariable-adjusted odds of spontaneous preterm birth (sPTB) (OR 0.81; 95% CI 0.70–0.94) (49).

In contrast, Black and African American women have higher relative abundances of *L. iners* and lower levels of *L. crispatus* than Caucasian women (49). This pattern is clinically significant because *L. iners* has not shown the same protective associations against PTB as *L. crispatus* across multi-cohort analyses (48). Notably, a British cohort found unexpectedly frequent dominance by *L. jensenii*, which was observed predominantly among Asian and Caucasian women (50). The complete absence of *L. gasseri* (CST II) in samples from Black women in this cohort further highlights population-specific differences in *Lactobacillus* species representation (50).

#### Geographic variations within populations of African descent

Importantly, geographic location introduces variation even within populations of similar ancestry. A meta-analysis comparing Nigerian and African American cohorts found significant differences in dominant taxa: *Atopobium* was more abundant in Nigerian samples, whereas *Sneathia* was more abundant in African American samples (51). Mean alpha-diversity values were 2.9 ± 0.09 for Nigerian women versus 3.3 ± 0.09 for African American women (*P* = 0.10), and taxon stability during pregnancy differed significantly between these groups (pADF <0.001) (51). These findings emphasize that biogeography and local environmental factors contribute to microbial composition independently of broad racial categorization (1, 51, 52).

#### CST distribution across populations

The traditional five-CST classification system (Fig. 1) reveals substantial population-level variation in CST prevalence, yet high-resolution taxon-level analyses often show stronger associations with pregnancy outcomes than CST categories alone (48). CST I (*L. crispatus*-dominated) is more prevalent in Caucasian and European populations and consistently associated with lower PTB risk across multiple studies (48, 49). CST III (*L. iners*-dominated) is more abundant in Black women and linked to higher alpha-diversity profiles in these populations, though *L. iners* itself has not demonstrated consistent protection against PTB (49). CST V (*L. jensenii*-dominated) was observed predominantly in Asian and Caucasian women in a British pregnant cohort, highlighting the ethnic specificity of this community type (50). CST IV (diverse anaerobic communities) is more common in Black and some African-descent populations, with specific anaerobic taxa varying by geography and ethnicity (51, 53, 54). Importantly, ethnic signatures affect not only CST prevalence but also the evolutionary dynamics and genetic diversity of constituent strains, with differences in nucleotide diversity and pN/pS ratios across Lactobacillaceae and non-Lactobacillaceae by ethnicity (55). These disparities indicate that CST distributions and their risk implications differ across populations, and that relying solely on standard CST classification can obscure population-specific taxa or subspecies signals linked to outcomes (48, 55).

#### Species and subspecies-level variation

Recent high-resolution metagenomic studies have revealed that associations between bacterial genera and pregnancy outcomes are often driven by specific subspecies or clades that vary in prevalence across populations. For example, associations between the genus *Gardnerella* and PTB are driven by specific subspecies or clades, and patterns of co-occurrence with *L. crispatus* (mutual exclusivity) and *L. iners* (frequent coexistence) have been documented (48, 53). The number of *Gardnerella* clades within an individual sample is associated with increased microbial load, and both clade richness and microbial load vary across cohorts, suggesting that community-level ecology and sampling location contribute to the observed differences (53). Meta-analytic evidence across PTB data sets has identified taxa such as *Finegoldia, Haemophilus*, and *Lactobacillus* species, as well as functional pathways, that vary by race/ethnicity and location, suggesting that PTB etiology and biomarker performance differ across populations (54). These findings underscore the importance of subspecies-level characterization in understanding population-specific microbiome-outcome relationships.

### Environmental factors

Environmental and behavioral factors interact with host genetics to shape vaginal microbiome composition during pregnancy and to modify associations between microbiome profiles and pregnancy outcomes. Several key environmental influences have been identified.

#### Socioeconomic status and social determinants

Socioeconomic variables correlate with microbiome differences and have been proposed as environmental drivers of ancestry-linked shifts in pregnancy microbiomes (56–58). These socioeconomic factors may operate through multiple pathways, including differential access to healthcare, nutritional status, exposure to stress, and living conditions. The complex interplay between socioeconomic status and microbiome composition suggests that observed racial and ethnic differences in the vaginal microbiome may be partially mediated by social determinants of health rather than solely by genetic ancestry (59).

#### Geographic and biogeographic influences

Geographic location independently shapes vaginal microbiome composition beyond host genetics or ethnicity (50, 51). As noted previously, distinct geographic populations (e.g., Nigerian versus African American women) show different dominant taxa (*Atopobium* versus *Sneathia*) and distinct longitudinal stability patterns during pregnancy, underscoring location as an independent modifier of microbial composition (51). These biogeographic effects may reflect local environmental exposures, dietary patterns, climate, or region-specific strains of vaginal bacteria. Understanding these geographic influences is essential for developing locally relevant screening tools and interventions. Summaries of the key metagenomic studies on pregnant and non-pregnant vaginal microbiome are shown in Table 5.

**TABLE 5:** Summary of key metagenomic studies on pregnant and non-pregnant vaginal microbiome

Population/country/ethnicity	Sample size	Sequencing methods	Major finding in pregnant/non-pregnant vaginal microbiome	Respective reference
North America/Caucasian and Black women	144 reproductive-age women	Terminal restriction fragment length polymorphism of 16S rRNA genes and phylogenetic analysis of dominant 16S rRNA gene sequences	Eight major vaginal communities were identified. Significant racial differences in community distribution were observed. Non-*Lactobacillus*-dominant communities were more frequent in Black than in Caucasian women. Black women have communities not dominated by *Lactobacillus* and enriched with *Atopobium* and *Clostridiales*. Communities with multiple *Lactobacillus* species were common in Caucasian women compared with Black women.	(20)
Reproductive age North American/White, Black, Hispanic, Asian	396 asymptomatic women	Pyrosequencing of barcoded 16S rRNA genes	Five groups in community: Four *Lactobacillus*-dominated (*L. iners, L. crispatus, L. gasseri,* and *L. jensenii*) and one diverse anaerobic-dominated group. Community distribution differed significantly by ethnicity. Vaginal pH was higher in Hispanic and Black women compared to Asian and White women. Correlated phylotypes were associated with high or low Nugent scores, which is a BV diagnosis factor.	(1)
Japanese women/Japan/Asian	73 asymptomatic Japanese women (18–45 years)	Terminal restriction fragment analysis of 16S rRNA genes and phylogenetic analysis of cloned 16S rRNA gene sequences	Vaginal community types in Japanese women were similar to those of White and Black women. Most communities were *Lactobacillus*-dominated. Mixed-*Lactobacillus* communities were common in Japanese and White, but rare in Black.	(60)
Healthy pregnancy and non-pregnant/USA	24 pregnant women (68 samples, 18–40 weeks gestation); 60 non-pregnant women (301 samples)	16S rRNA gene sequencing (V5–V3 region) using 454 FLX Titanium platform	Pregnancy reduced diversity and richness of vaginal microbiome. The dominance of the pregnancy vaginal microbiome is *Lactobacillus* species (*L. iners, L. crispatus, L. jensenii,* and *L. johnsonii*), with differences across vaginal subsites and gestational age.	(61)
Reproductive age/USA	22 pregnant women (38–42 weeks); 32 non-pregnant women	16S rRNA gene sequencing using pyrosequencing	Pregnant women had higher abundance of *Lactobacillus* vaginalis, *L. crispatus, L. gasseri,* and *L. jensenii* and lower abundance of 22 phylotypes compared to non-pregnant women. BV-associated CST IV-A/B were less frequent in pregnancy. Microbiota stability in the vagina was higher in pregnancy.	(3)
Pregnant women/Chinese/Asian	34 pregnant women	16S rRNA tag sequences	High homogeneity of vaginal microbiome across cervix, posterior fornix, and vaginal canal within individuals. The vaginal microbiome variation among women during trimester T1 is larger than that of non-pregnant women and other trimesters. Postpartum microbiomes are notably different from gestational microbiomes.	(62)
European population/mixed British cohort/Asian, Caucasian, Black	42 pregnant women (8–12, 20–22, 28–30, 34–36 weeks; 6 weeks postpartum)	MiSeq sequencing of 16S rRNA gene amplicon	During pregnancy, the vaginal microbiome was characterized by *Lactobacillus* dominance and low alpha-diversity. In the postpartum period, the microbiome composition shifted dramatically to reduced *Lactobacillus* dominance and increased alpha-diversity, independent of ethnicity. *L. jensenii* was predominantly observed in Asian and Caucasian women, whereas *L. gasseri* was absent in Black women.	(50)
Pregnant women aged 18 years or older/USA/nonspecific	49 pregnant women (15 preterm); 40 women with weekly longitudinal sampling; validation cohort: 9 women (four preterm)	16S rRNA gene-based bacterial taxonomic analysis	Vaginal microbiota remained stable during pregnancy. *Lactobacillus*-poor CST IV was inversely correlated with gestational age and associated with increased risk of preterm birth, especially when accompanied by *Gardnerella* or *Ureaplasma*. Postpartum, most women experienced shifting decreased *Lactobacillus* and increased diverse anaerobe, lasting up to 1 year.	(32)
Women attending antenatal clinics at Mount Sinai Hospital (Toronto, ON, Canada)/multiethnicity	182 pregnant women (11–16 weeks of gestation); 310 pregnant women	Pyrosequencing of the cpn60 universal target region	CST profiles were similar between pregnant and nonpregnant women; healthy pregnant women had lower richness and diversity, lower prevalence of *Mycoplasma* and *Ureaplasma*, high bacterial load, and greater *Lactobacillus* abundance compared to non-pregnant women	(19)
Pregnant women at risk of preterm birth; United Kingdom	161 pregnant women at risk of preterm birth; progesterone subgroup: 25 treated vs 42 controls	16S rRNA gene sequencing	*L. iners* dominance at 16 weeks was associated with a short cervix and preterm birth less than 34 weeks. *L. crispatus* dominance was highly predictive of term birth. Cervical shortening and preterm birth were not associated with any vaginal dysbiosis. Vaginal progesterone did not alter microbiota structure.	(30)
First-trimester pregnant women/USA	155 first-trimester pregnant women (52 first conception; 26 prior abortions; 77 prior birth)	16S rRNA gene sequencing (V1–V3 region)	*L. crispatus* dominance was the highest in women with first conception (76.4%) and decreased with prior abortion (50%) and prior birth (22.2%). *L. iners* dominance was more frequent in women with prior abortion or birth. Also, the dominance was associated with a history of spontaneous abortion. *Gardnerella* dominance increased with prior pregnancy history.	(63)
USA; racially distinct cohorts	308 subjects across cohorts	16S rRNA gene amplicon sequencing data	Lower *L. crispatus* and higher *Gardnerella vaginalis* (G2 clade) associated with preterm birth in low-risk cohort; associations weaker in high-risk Black cohort; no direct pregnant vs. non-pregnant, but implies pregnancy instability	(48)
USA; multiracial/ethnic (African American, Caucasian, Hispanic, Asian)	613 pregnant women (large multi-ethnic cohort)	16S rRNA gene sequencing (V4)	Significant racial-ethnic diversity in vaginal microbiome dynamics during pregnancy; non-*Lactobacillus*-dominant communities are more frequent in African American/Hispanic women, and certain CST transitions are associated with preterm birth risk.	(11)
USA	Cohort of 2,000 pregnant women (107 spontaneous preterm birth [sPTBX] and 432 delivering at term)	16S rRNA gene sequencing	Cervicovaginal microbiota and local immune response modulate preterm delivery risk. Dysbiosis (low *Lactobacillus*) in pregnancy is linked to inflammation and increased risk of spontaneous preterm birth	(56)
Healthy pregnant women/ Russian	22 first-trimester pregnant women	16S rRNA sequencing (NGS)	*L. iners* dominated microbiome were predominant in early pregnancy. Cervical microbiomes have higher alpha diversity than cervicovaginal samples. Patients with soil- or animal-associated bacteria might relate to rural origin of patients.	(64)

## FUTURE RESEARCH

Many studies examining changes in the vaginal microbiome during pregnancy are limited by cross-sectional designs that recruit both pregnant and non-pregnant women simultaneously (19). This approach complicates attributing observed differences to pregnancy rather than to individual variability or confounding factors. To establish causal relationships regarding microbiome changes, large-scale longitudinal studies that follow women over time are required. These studies would clarify the specific effects of pregnancy on microbiome composition and function and track the stability and transitions of microbial communities.

Davidson et al. (65) argue that current sampling, DNA extraction, and sequencing methods may miss critical aspects of the vaginal microbiome, thereby limiting the accuracy of research findings (65). Addressing these technological and methodological limitations is crucial for advancing understanding in this field. For instance, although 16S rRNA gene sequencing is widely used, it is prone to primer bias and lacks sufficient resolution to detect fine-scale microbial differences. Adopting broader approaches, such as metagenomics, alongside standardized protocols will improve the classification of community types and facilitate cross-study comparisons, ultimately supporting more robust and reliable outcomes.

A critical direction for future research is to determine whether interventions targeting the vaginal microbiome can improve pregnancy outcomes. Although previous studies have linked elevated *Lactobacillus* levels to stable pregnancies and there is growing interest in probiotics and prebiotics, the individuality of each woman’s microbiome suggests that universal treatments are improbable (66). Progress in this field requires a shift from identifying associations to elucidating causal mechanisms.

Establishing the vaginal microbiome’s influence on pregnancy outcomes requires moving from association-based studies to those demonstrating causation. Future research should investigate the specific roles of individual microbes and their effects on immune function, vaginal barrier integrity, and inflammatory processes. Furthermore, examining interactions between the vaginal microbiome and other body sites, such as the gut or oral cavity, is essential for a comprehensive understanding of system-wide changes during pregnancy.

## CONCLUSION

This review highlights that pregnancy is strongly associated with distinct changes in the vaginal microbiome. Compared with the non-pregnant state, pregnancy is linked to reduced microbiome variability and increased dominance of *Lactobacillus* species. These changes reflect a shift toward a more protective environment for the fetus. In contrast, dominance by anaerobic taxa has frequently been associated with adverse reproductive outcomes. Importantly, vaginal microbiome composition during pregnancy varies across populations. For example, racial and ethnic groups, people living in different geographic locations, and individuals exposed to distinct environmental factors often have different community structures, dominant *Lactobacillus* species, and stability in their vaginal microbiomes. Recognizing these population-level patterns helps explain varying pregnancy outcomes. It also underscores the need to question the sole reliance on community-state-type classifications, which may mask clinically relevant, population-specific microbial signatures.

Advances in 16S rRNA sequencing, next-generation sequencing, and shotgun metagenomics have improved understanding of the vaginal microbiome. These techniques provide higher-resolution taxonomic and functional insights. Associations between the vaginal microbiome and pregnancy outcomes often reflect species-level differences rather than broad taxonomic groups. However, limitations in current analytical techniques and study designs still restrict the ability to identify causal relationships. This review supports associations among vaginal microbiome composition, pregnancy status, and maternal–neonatal health outcomes. It also highlights important gaps in understanding causality. Future research should prioritize longitudinal studies across diverse populations to clarify temporal dynamics. Researchers should address confounding factors and test whether targeted microbiome-based interventions can improve pregnancy outcomes. Advancing mechanistic insight will be essential for progress in this field.

## LIMITATIONS OF THIS REVIEW

This review has several limitations. First, most included studies are observational and cross-sectional, limiting causal inference regarding the relationship between vaginal microbiome composition and pregnancy outcomes. Second, heterogeneity in sequencing platforms, targeted 16S regions, bioinformatic pipelines, and CST classification methods complicates direct comparison across studies. Third, many populations—particularly low-resource settings—remain underrepresented, which may bias conclusions toward well-studied cohorts from North America and Europe. Finally, most studies focus primarily on bacterial communities, with limited integration of viral, fungal, and functional metagenomic data. These limitations highlight the need for standardized methodologies and more inclusive, longitudinal research designs.

## RATIONALE FOR THIS REVIEW

This article addresses important gaps in understanding how the vaginal microbiome changes during pregnancy and how these changes influence maternal and neonatal health outcomes. Although previous studies have shown that pregnancy is associated with reduced microbial diversity and increased Lactobacillus dominance, much of the existing evidence comes from European and North American populations. Consequently, the applicability of these findings to diverse racial, ethnic, and geographic groups remains limited. Underrepresented populations are rarely included in vaginal microbiome research. Additionally, many earlier studies relied primarily on 16S rRNA sequencing, which provides limited functional and taxonomic resolution. Recent advances in next-generation and shotgun metagenomic sequencing enable more comprehensive characterization of microbial communities. This review synthesizes findings from these advanced methodologies to provide an updated synthesis of pregnancy-associated vaginal microbiome dynamics. It also examines community state types, gestational timing, and population-specific microbial patterns. By linking microbiome variation to clinical pregnancy and neonatal outcomes, this article highlights potential contributors to health disparities. Ultimately, this work aims to guide future longitudinal research and inform the development of targeted microbiome-based interventions to improve maternal and neonatal health.
